# Projected climate change impacts in rainfall erosivity over Brazil

**DOI:** 10.1038/s41598-017-08298-y

**Published:** 2017-08-15

**Authors:** André Almagro, Paulo Tarso S. Oliveira, Mark A. Nearing, Stefan Hagemann

**Affiliations:** 1 Federal University of Mato Grosso do Sul, CxP 549, Campo Grande, MS 79070-900 Brazil; 2 USDA-ARS, Southwest Watershed Research Center, 2000 E. Allen Rd., Tucson, AZ 85719 United States; 3 Max-Planck-Institut für Meteorologie, Bundesstr. 53, 20146 Hamburg, Germany

## Abstract

The impacts of climate change on soil erosion may bring serious economic, social and environmental problems. However, few studies have investigated these impacts on continental scales. Here we assessed the influence of climate change on rainfall erosivity across Brazil. We used observed rainfall data and downscaled climate model output based on Hadley Center Global Environment Model version 2 (HadGEM2-ES) and Model for Interdisciplinary Research On Climate version 5 (MIROC5), forced by Representative Concentration Pathway 4.5 and 8.5, to estimate and map rainfall erosivity and its projected changes across Brazil. We estimated mean values of 10,437 mm ha^−1^ h^−1^ year^−1^ for observed data (1980–2013) and 10,089 MJ mm ha^−1^ h^−1^ year^−1^ and 10,585 MJ mm ha^−1^ h^−1^ year^−1^ for HadGEM2-ES and MIROC5, respectively (1961–2005). Our analysis suggests that the most affected regions, with projected rainfall erosivity increases ranging up to 109% in the period 2007–2040, are northeastern and southern Brazil. Future decreases of as much as −71% in the 2071–2099 period were estimated for the southeastern, central and northwestern parts of the country. Our results provide an overview of rainfall erosivity in Brazil that may be useful for planning soil and water conservation, and for promoting water and food security.

## Introduction

Soil erosion has generated environmental, social and economic damage worldwide^1^. Recent studies indicate that the world’s food production needs to increase by 60% to 110% to meet global demand growth^2, 3^ and that soil erosion will increase in 21^st^ century due to climate change^4–6^. Therefore, one of the greatest challenges of this century is to promote water and food security by efficient agricultural productivity and reduction of soil erosion^7^.

Agribusiness is the main economic resource of Brazil. Currently, the country is one of the world’s largest producers and exporters of grain and beef ^8^, and the majority of those products are produced in the Cerrado (Central-West region). In 2016, agribusiness was responsible for 23% of Gross Domestic Product (GDP) and 50% of exports of Brazil (China, European Union, and the United States being the major importers)^9^. Therefore, a better understanding of climate change impacts on soil erosion processes is also important to the Brazilian economy.

Rainfall erosivity is the potential of rainfall to cause soil erosion by raindrop impact and surface wash out when infiltration capacity is exceeded. It is commonly represented by the R-factor in soil erosion prediction models such as Universal Soil Loss Equation (USLE)^10^ and it revised version (RUSLE)^11^. As rainfall is the driving force of water erosion, the rainfall erosivity is an important factor in the USLE and its revised versions^12^. It is a crucial parameter for soil erosion risk assessment that considers different future land use and climate change scenarios^13^, and for proposing conservation practices^14^. The R-factor is also an important parameter for assessing the risk of soil erosion under global warming conditions because it is highly influenced by a changing climate^13, 15, 16^.

Climate change can alter rainfall erosivity due to alteration of rainfall patterns^17^. The increase in global mean temperature generates an increase in the moisture retention capacity of the atmosphere that is in the order of 7% per degree Celsius^18^. The addition of water vapor in the atmosphere influences climate circulation patterns, thus modifying the intensity, frequency and incidence of extreme rainfall events. In warm climates, such as in Brazil, temperature and extreme rainfall events will increase more significantly than in many other regions around the world^18, 19^.

Future potential changes in rainfall erosivity can be projected using precipitation data from General Circulation Models (GCM)^20^ under different greenhouse gases emissions scenarios that follow the various Representative Concentration Pathways (RCPs) of the Intergovernmental Panel on Climate Change 5^th^ Assessment Report^21^. However, GCMs usually provide global data at a rather coarse resolution (grid size about 100–200 km) so that for climate change impact studies, it is usually necessary to downscale GCM simulations using Regional Climate Models (RCM) to provide spatially more detailed data and resolve regional or local forcings^22^.

To support strategic climate change studies and the Brazilian Third National Communication to the United Nations Framework Convention on Climate Change, National Institute for Space Research (INPE) carried out four sets of downscaling simulations using the Eta RCM forced by medium (RCP 4.5) and high (RCP 8.5) emission scenarios of HadGEM2-ES and MIROC5 GCMs. The choice of these two models was based on their satisfactory simulation of precipitation and atmospheric circulation over South America, and on the easy data availability made possible by the British Atmospheric Data Centre and National Institute for Environmental Studies of Japan^23, 24^. The resulting data is the most advanced and refined dataset for climate change studies in South America and was adopted as the official data for the Brazilian National Adaptation Plan for Climate Change^25–27^.

Studies of rainfall erosivity have been conducted for Brazil since the 1980s. However, most of these studies have been done using short rainfall time series^28^ and do not assess the impacts of climate change. In a review of rainfall erosivity in Brazil, Oliveira *et al*.^28^ reported that 85% of the analyzed papers were developed using a time series shorter than 20 years; in other words, only 15% of these studies used the minimum series suggested for USLE/RUSLE applications^11^. At the basin scale, climate change and its impacts on rainfall erosivity have already been investigated in many parts of the world, such as Australia^29^, Brazil^30^, China^31, 32^, England^33^, India^17^, Japan^34^, Thailand^35^ and the United States^20^. However, to our knowledge there has not been an investigation of the impacts of projected climate change in rainfall erosivity across Brazil, as has already been done for the United States^1, 36^ and Europe^37^. These studies are needed to help guiding future land use, and soil and water conservation plans.

The objective of this study is to assess the climate change influence on rainfall erosivity across Brazil. We used observed rainfall data (1980–2013) and downscaled climate model output (baseline, 1961–2005, and projected, 2007–2099) from the GCMs HadGEM2-ES and MIROC5 forced by RCP4.5 and RCP8.5 scenarios. This study provides an overview (past and projections) of the regions of Brazil where projected changes in rainfall erosivity are the greatest and discusses the potential impacts on croplands across the country.

## Results and Discussion

### Projected impacts of climate change on rainfall erosivity

**Figure 1:**
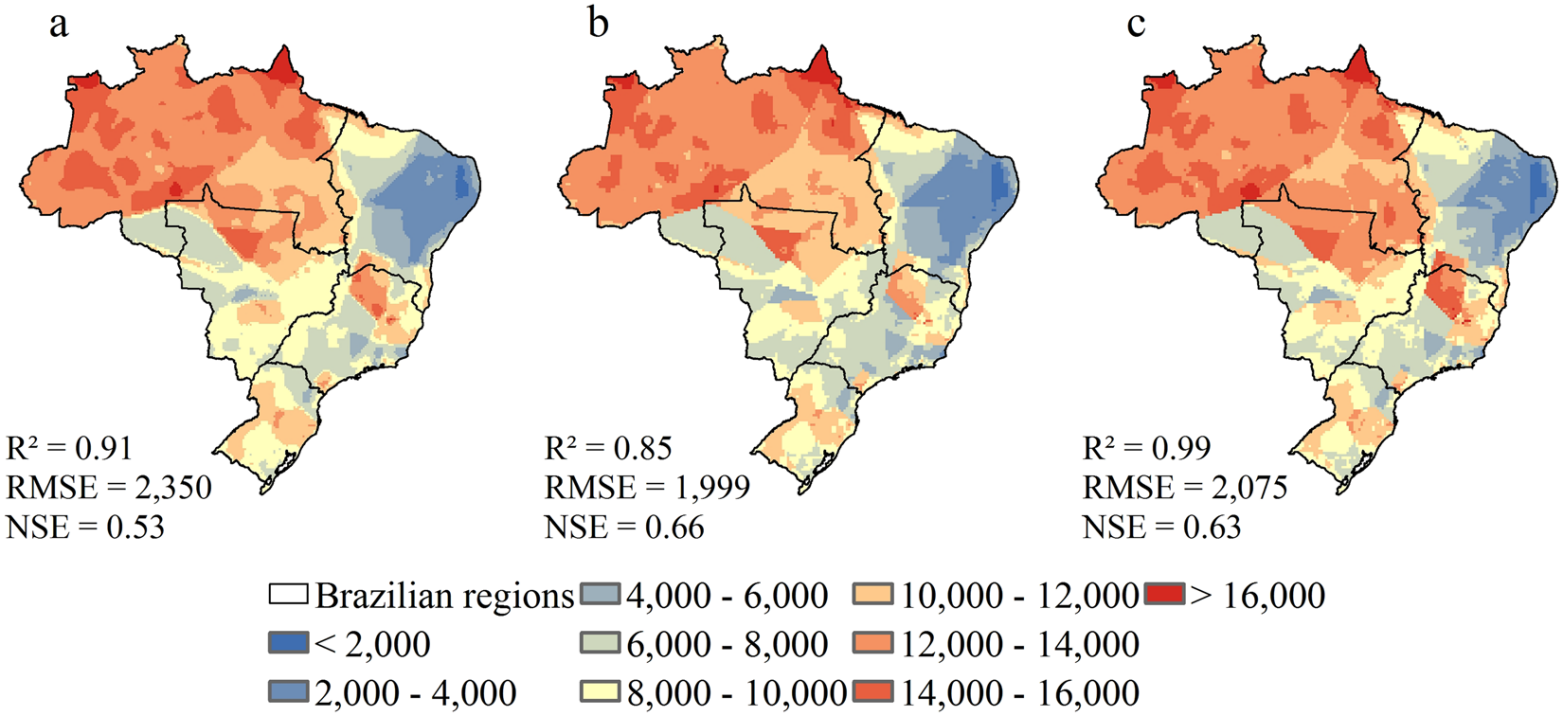
Rainfall erosivity for Brazilian regions. (**a**) Observed rainfall erosivity (1980–2013); (**b**) HadGEM2-ES estimations for baseline (1961–2005); (**c**) MIROC5 estimations for baseline (1961–2005). Values are in MJ mm ha^−1^ h^−1^ year^−1^. Maps created with ESRI ArcGIS 10.1 (www.esri.com).

**Figure 2:**
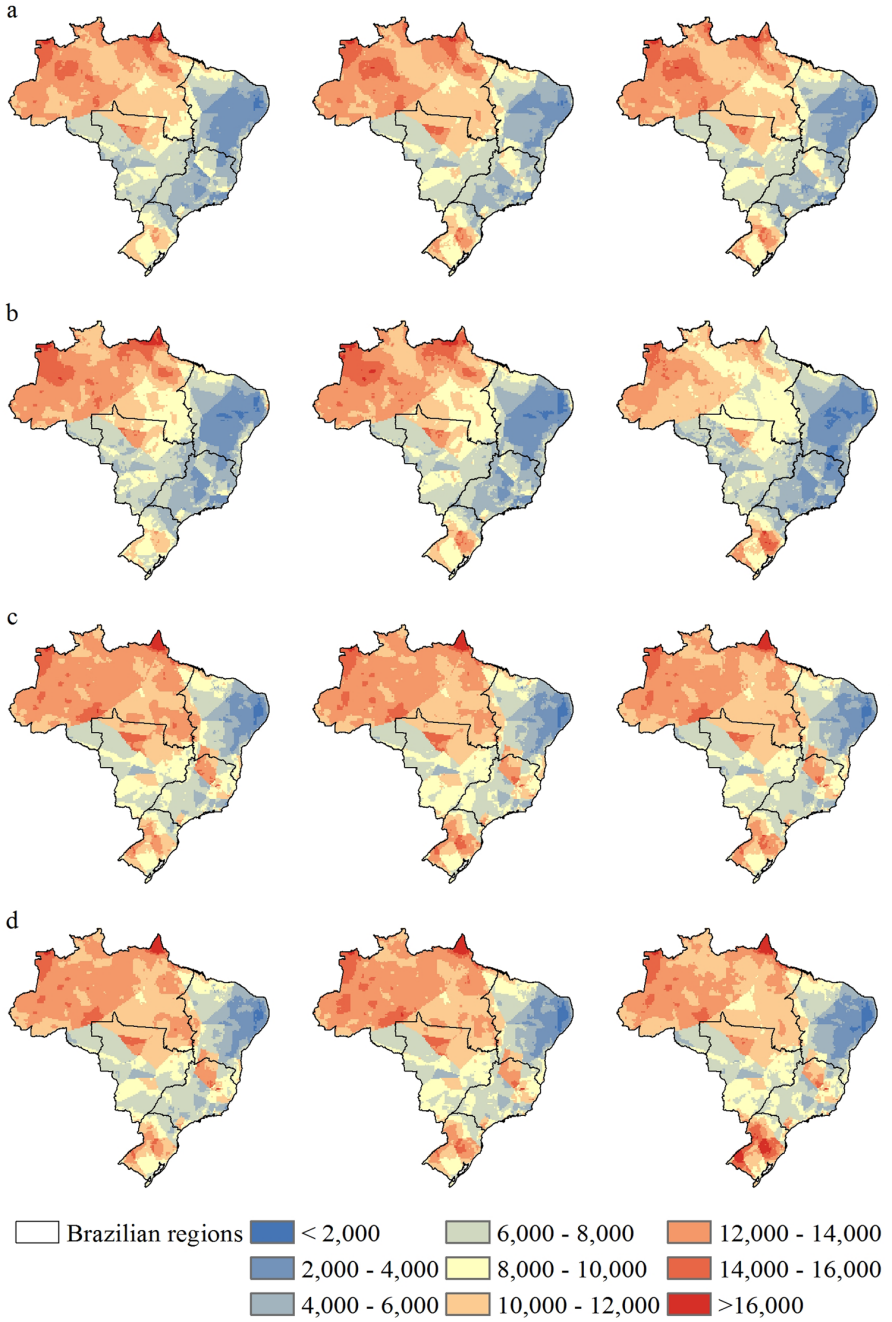
Estimated rainfall erosivity for Brazilian regions through 21^st^ century. (**a**) RCP4.5 scenario of HadGEM2-ES model for three periods (2007–2040, 2041–2070 and 2071–2099). (**b**) RCP8.5 scenario of HadGEM2-ES model for three periods (2007–2040, 2041–2070 and 2071–2099). (**c**) RCP4.5 scenario of MIROC5 model for three periods (2007–2040, 2041–2070 and 2071–2099). (**d**) RCP8.5 scenario of MIROC5 model for three periods (2007–2040, 2041–2070 and 2071–2099). Values in MJ mm ha^−1^ h^−1^ year^−1^. Maps created with ESRI ArcGIS 10.1 (www.esri.com).

**Figure 3:**
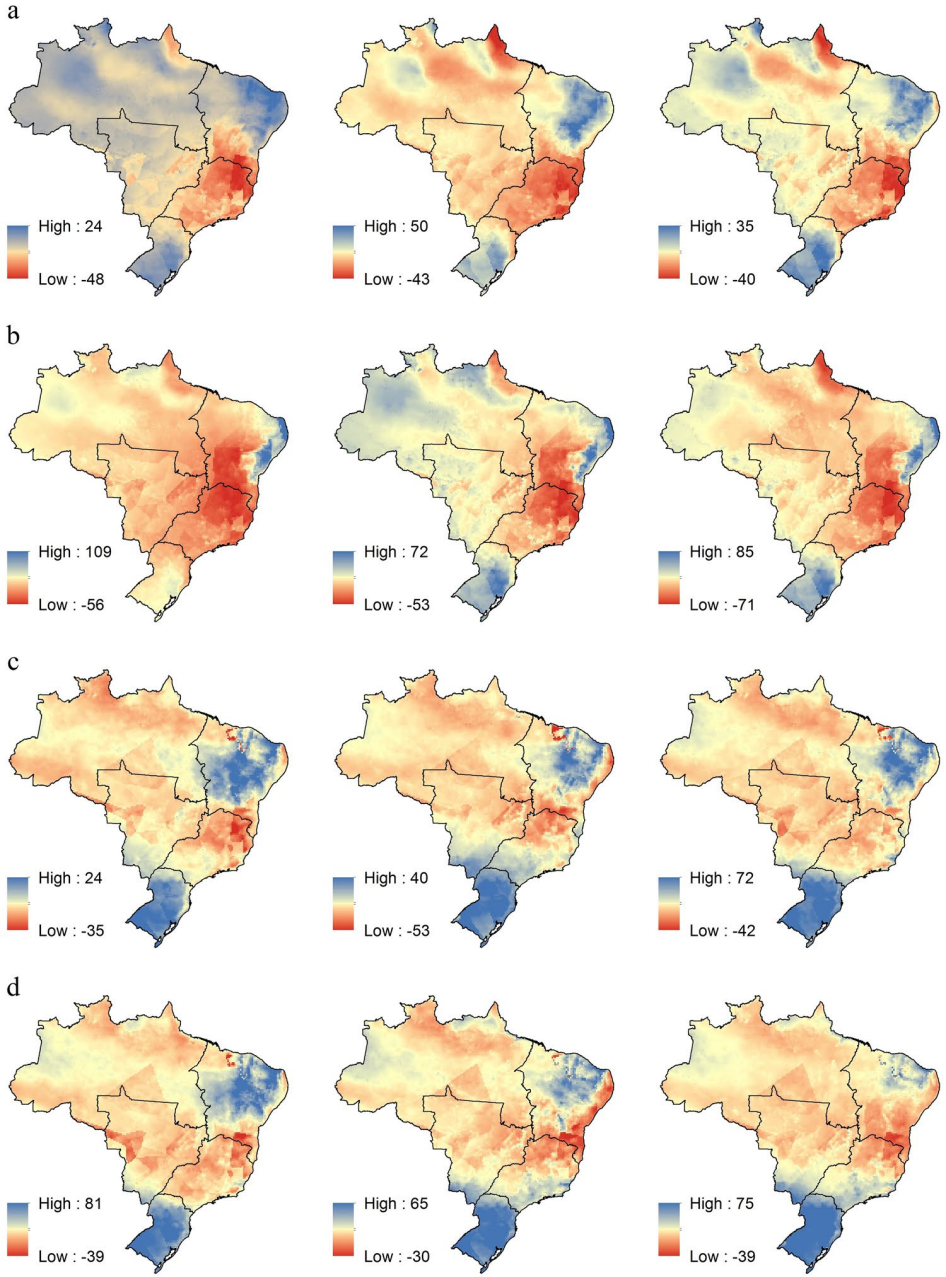
Relative difference between projections and baseline rainfall erosivity for Brazilian regions. Red colors represent decreasing trends and blue colors represents increasing trends for: (**a**) RCP4.5 scenario of HadGEM2-ES model for three periods (2007–2040, 2041–2070 and 2071–2099). (**b**) RCP8.5 scenario of HadGEM2-ES model for three periods (2007–2040, 2041–2070 and 2071–2099). (**c**) RCP4.5 scenario of MIROC5 model for three periods (2007–2040, 2041–2070 and 2071–2099). (**d**) RCP8.5 scenario of MIROC5 model for three periods (2007–2040, 2041–2070 and 2071–2099). Maps created with ESRI ArcGIS 10.1 (www.esri.com).

Figures 1 and 2 show the spatial variability and Tables 1 and 2 present statistics of the observed (1980–2013), baseline (1961–2005) and projected (2007–2099) rainfall erosivity for each Brazilian region. In addition, Fig. 3 shows the relative difference between each projected scenario and models’ baseline. Observed erosivity estimated was 10,437 ± 3,409 MJ mm ha^−1^ h^−1^ year^−1^ (1,708 to 21,767 MJ mm ha^−1^ h^−1^ year^−1^). For the baseline, R-factor values ranged from 1,580 to 24,708 MJ mm ha^−1^ h^−1^ year^−1^, with an average of 10,089 ± 3,489 MJ mm ha^−1^ h^−1^ year^−1^, for HadGEM2-ES. For MIROC5, a mean rainfall erosivity was 10,585 ± 3,420 MJ mm ha^−1^ h^−1^ year^−1^, ranging from 1,666 to 23,417 MJ mm ha^−1^ h^−1^ year^−1^. The greatest values occur in the North region and the least in the Northeast for observed data and both models. Our results are in agreement with previous studies of rainfall erosivity over Brazil^14, 28, 38, 39^ and statistical tests yielded satisfactory performance of our estimations.

**Table 1:** Basic statistics of rainfall erosivity in Brazilian regions.

		South	Southeast	North	Northeast	Central-West
Observed (1980–2013)	Min.	5702	3981	6451	1708	5438
	Max.	12747	16198	21676	14156	15678
	Mean	9362	8876	13118	6126	9618
	STD	1567	2192	1858	2841	2146
Baseline (1961–2005)	Min.	5212	3485	6209	1580	5170
	Max.	12647	15520	24708	13463	15213
	Mean	9038	8028	12885	5858	9192
	STD	1628	2126	2016	2720	2215
RCP 4.5 (2007–2040)	Min.	4155	2368	5813	1750	4449
	Max.	13371	10572	18067	11728	14408
	Mean	8674	5615	11847	5201	7960
	STD	2039	1505	1890	2337	2129
	Change (%)	−4	−30	−8	−11	−13
RCP 4.5 (2041–2070)	Min.	4650	2481	5905	1894	4737
	Max.	15470	11442	17446	12010	14908
	Mean	9664	6392	12004	5806	8524
	STD	2339	1701	1838	2326	2140
	Change (%)	7	−20	−7	−1	−7
RCP 4.5 (2071–2099)	Min.	4612	2571	5885	1740	4734
	Max.	15774	11438	17457	11536	14924
	Mean	9728	6279	11894	5453	8424
	STD	2372	1704	1906	2377	2064
	Change (%)	8	−22	−8	−7	−8
RCP 8.5 (2007–2040)	Min.	4246	2184	5184	1538	4474
	Max.	13349	10693	18450	13635	14572
	Mean	8480	5336	11917	4885	7816
	STD	1919	1400	2202	2327	2094
	Change (%)	−6	−34	−8	−17	−15
RCP 8.5 (2041–2070)	Min.	4538	2282	5680	1601	4498
	Max.	16054	11536	19012	11285	14842
	Mean	9482	5639	11952	4796	8091
	STD	2369	1511	2218	2272	2040
	Change (%)	5	−30	−7	−18	−12
RCP 8.5 (2071–2099)	Min.	4160	1693	5337	1483	4234
	Max.	17627	10680	18936	12342	14482
	Mean	9991	4729	10345	4399	7522
	STD	2855	1460	2023	2044	1803
	Change (%)	11	−41	−20	−25	−18

Observed and estimated by HadGEM2-ES model. Minimum, maximum, mean, standard deviation (MJ mm ha^−1^ h^−1^ year^−1^) and projected change in relation to baseline (%).

**Table 2:** Basic statistics of rainfall erosivity in Brazilian regions.

		South	Southeast	North	Northeast	Central-West
Observed (1980–2013)	Min.	5702	3981	6451	1708	5438
	Max.	12747	16198	21676	14156	15678
	Mean	9362	8876	13118	6126	9618
	STD	1567	2192	1858	2841	2146
Baseline (1961–2005)	Min.	5381	3672	6345	1666	5407
	Max.	13281	18449	23417	15489	15990
	Mean	9229	9097	13167	6511	9816
	STD	1668	2684	1846	3018	2389
RCP 4.5 (2007–2040)	Min.	5547	3460	5932	1653	5200
	Max.	14752	16789	22489	14294	15142
	Mean	10026	8330	12345	6498	9262
	STD	2027	2361	1763	2846	2178
	Change (%)	9	−8	−6	0	−6
RCP 4.5 (2041–2070)	Min.	6130	3683	5894	1595	5190
	Max.	16395	17448	21994	13611	14867
	Mean	10759	8632	12259	6301	9287
	STD	2316	2284	1781	2725	2111
	Change (%)	17	−5	−7	−3	−5
RCP 4.5 (2071–2099)	Min.	6081	3688	5787	1651	4896
	Max.	16122	17783	22161	13861	14924
	Mean	10605	8354	12106	6329	8924
	STD	2238	2455	1807	2674	2127
	Change (%)	15	−8	−8	−3	−9
RCP 8.5 (2007–2040)	Min.	5598	3399	5796	1686	4926
	Max.	15532	16862	21145	14352	14736
	Mean	10107	8121	12138	6331	8892
	STD	2211	2417	1752	2720	2174
	Change (%)	10	−11	−8	−3	−9
RCP 8.5 (2041–2070)	Min.	6028	3758	5980	1439	5054
	Max.	16244	17734	21928	13703	15191
	Mean	10563	8486	12370	6197	9164
	STD	2220	2290	1832	2753	2125
	Change (%)	14	−7	−6	−5	−7
RCP 8.5 (2071–2099)	Min.	6766	3885	5584	1423	4853
	Max.	19815	16943	23137	14101	14515
	Mean	12263	8289	11894	5789	8885
	STD	2997	2151	1904	2625	1991
	Change (%)	33	−9	−10	−11	−9

Observed and estimated by MIROC5 model. Minimum, maximum, mean, standard deviation (MJ mm ha^−1^ h^−1^ year^−1^) and projected change in relation to baseline (%).

In the North region, we calculated mean values of rainfall erosivity of 12,885 and 13,167 MJ mm ha^−1^ h^−1^ year^−1^ during the baseline period for HadGEM2-ES and MIROC5, respectively. From HadGEM2-ES and both scenarios, we note a decrease of −7% to −20% in the mean rainfall erosivity in this region with a slight increase in the western part and a strong increase in the northern part of this region. We also found similar results using MIROC5 for both scenarios, with decreases ranging from −6% to −10%. The greatest decrease in rainfall erosivity is expected for the end of the 21^st^ century (2071–2099 period), using the RCP8.5 scenario.

The average rainfall erosivity in the Northeast for the baseline period was 6,511 MJ mm ha^−1^ h^−1^ year^−1^ and 5,858 MJ mm ha^−1^ h^−1^ year^−1^ for MIROC5 and HadGEM2-ES, respectively. This region has the lowest mean rainfall erosivity of Brazilian regions. Furthermore, we note a great range in erosivity for this region, likely because it includes a semiarid area called Caatinga (low values) and a part of the Brazilian coast (high values). The HadGEM2-ES scenarios (RCP4.5 and RCP8.5) project decreases from −1% to −25% in rainfall erosivity for the future periods, with accentuated values for the end of the 21^st^ century. The MIROC5 results yield changes from zero to −11% during the century for the two emissions scenarios. Although the average values indicate a decrease, there are areas where an increase in rainfall erosivity is projected. The RCP4.5 scenario from both models and the RCP8.5 scenario from MIROC5 indicate that the central area of Northeast is expected to have the greatest increases, while the RCP8.5 scenario from HadGEM2-ES project an increase only in the coastal area.

In the Central-West region, HadGEM2-ES and MIROC5 yield, respectively, average rainfall erosivity values of 9,192 MJ mm ha^−1^ h^−1^ year^−1^ and 9,816 MJ mm ha^−1^ h^−1^ year^−1^ in the baseline period. Overall, we noted a decrease in the average rainfall erosivity in the Central-West region for all models and scenarios compared to the baseline. For both scenarios, MIROC5 projected decreases in erosivity varying from −5% to −9% (increases were projected only in the south of Mato Grosso do Sul state). HadGEM2-ES projected decreases by as much as −18% with the RCP8.5 emission scenario.

In the baseline period, the Southeast region has an estimated average rainfall erosivity of 8,028 and 9,097 MJ mm ha^−1^ h^−1^ year^−1^ for HadGEM2-ES and MIROC5, respectively. The HadGEM2-ES scenarios (RCP4.5 and RCP8.5) project a decrease in the mean rainfall erosivity of up to −20%, reaching −41% in the latter part of the century. Some areas of the Southeast can reach very low rainfall erosivity values only observed in the Northeast in baseline period. Similar to HadGEM2-ES, but in a lesser magnitude, the MIROC5 scenarios also projected a decrease in mean rainfall erosivity that ranged from −5% to −11% for the Southeast region during the 21^st^ century, but with increases in coastal areas by the middle of the century.

The South region had average baseline rainfall erosivities of 9,038 MJ mm ha^−1^ h^−1^ year^−1^ and 9,229 MJ mm ha^−1^ h^−1^ year^−1^ for HadGEM2-ES and MIROC5, respectively. The HadGEM2-ES scenarios projected a mean decrease ranging from −4% to −6% for the 2007–2040 period and an increase (5% to 11%) for the rest of the 21^st^ century. MIROC5 projected increases in the mean annual rainfall erosivity of more than 9%. In general, most scenarios projected a strong increase in rainfall erosivity over the South region. This increase has the potential to increase soil erosion rates, which may ultimately affect the productivity of primary croplands of the South region, which are responsible for a significant part of Brazilian agricultural production (see Supplementary Fig. S1).

The decrease in the rainfall erosivity over the North, Northeast, Central-West and Southeast regions and the increase for the South region projected by the scenarios may be consequences of the intensification of frequency and magnitude of the El Niño Southern Oscillation (ENSO), the anomalous warming in equatorial Pacific Ocean (Peru and Ecuador coast) which causes climatic variation on broad regions of the Earth. In Brazil, it causes negative anomalies in annual rainfall in the North, Northeast, Central-West and part of Southeast, and positive anomalies in the South^40, 41^, especially in the South America Summer Monsoon (SASM) regime^42^. The SASM takes place in the January, February, and March (JFM) period and is responsible for the rainy season in Brazil. Moreover, JFM contributes a great part of the annual rainfall erosivity in all Brazilian regions^43^. The ENSO also affects the South Atlantic Convergence Zone (SACZ), an elongated convective band originating in Amazon basin (North), extending toward the Southeast and protruding to the southern Atlantic Ocean, which is one of the main components of SASM^44^. Once that ENSO decreases the convective activity of the SACZ, a decrease in rainfall and rainfall erosivity is expected for regions from North to Southeast. On the other hand, the increase in rainfall erosivity projected for the central portion of Northeast can be explained by the intensification of the Intertropical Convergence Zone (ITCZ) – with a large magnitude in RCP 4.5 – which causes increases in rainfall amounts and extreme rainfall events in this part of Northeast, especially during the rainy season (summer)^45, 46^. In all cases RCP 8.5 projects drier conditions than RCP 4.5.

### Adaptation to the rainfall erosivity changes

More important than the projected impacts of climate change in rainfall erosivity is the identification of how the country can adapt to these changes by adopting strategies for decreasing vulnerabilities and increasing resilience. Public policies that aim for soil and water conservation must be encouraged and implemented at the level of the land managers. Our study provides important information about what we can expect in the future regarding the soil erosion potential over Brazil. The impacts of climate change for additional factors need to be assessed to provide a more effective and precise guidance of land-use and sustainable planning, such as the drought index, water availability, agricultural land aptitude and local infrastructure. Thus, allied with these additional types of information, our results may be useful for developing a soil and water conservation plan for Brazilian regions.

Investments will be necessary to implement conservation practices, sustainable agriculture and technological improvements to ensure an agricultural intensification (ensuring food security) while maintaining acceptable levels of soil losses^3, 47, 48^. Regions in which models indicate a decrease in mean rainfall erosivity tend to be more attractive for agricultural expansion, due to the consequent decrease in associated potential soil loss. Although there might be decreases in soil loss associated with decreases in rainfall erosivity, the conversion of natural land cover into croplands tends to increase soil loss rates by an order of magnitude or more, if not done with effective conservation practices^7, 49, 50^.

The projected decreases in mean rainfall erosivity in North and Northeast may be important for a new major frontier of agribusiness known as MATOPIBA. This area corresponds to parts of states located in the North (Tocantins) and Northeast (Maranhão, Piauí and Bahia) regions^51, 52^. MATOPIBA is one of the largest expanding agricultural frontiers in the world and a strategic area for the development of Brazilian economy^53^. All indications are that farmland expansion will continue in the MATOPIBA^3, 7^. At the same time, the Northeast is a more susceptible region to projected climatic changes^54^ and to its precarious conditions of infrastructure and available technology. An increase in rainfall erosivity may degrade soil fertility and water availability, increasing the vulnerability of smallholders and subsistence farmers^55^.

The South region may experience more intensive increases in rainfall erosivity in Brazil according to all considered projections. Soil conservation practices must be implemented in croplands, which cover more than 36% of total area of the region^56^. Conservation seeding techniques (e.g. no-tillage), agricultural crops that cover the soil surface and seeding in the dry season must be followed, and the riparian forest must be conserved to mitigate the impacts of the increase in rainfall erosivity on soil and water quality and availability.

The North, Central-West and Southeast regions are projected to experience decreases in rainfall erosivity in 21^st^ century, especially during the latter half. This will occur probably due to the reduction of SACZ activity which is responsible for a great part of the rainfall amounts in these regions. The decrease in rainfall erosivity will generate less soil loss rates in the regions, and it is projected that agricultural production in North (Amazon) and Central-West (Cerrado) will increase. Thus, more natural vegetation cover may be converted to croplands, as discussed previously by Oliveira *et al*.^57^ and Morton *et al*.^58^. The Southeast is the main producer of sugar cane in Brazil^59^ and our projections suggests that the decrease in rainfall erosivity may create the ideal conditions to continue the recent expansion in the culture of this crop. Previous studies indicated that Brazilian sugar cane production needs to increase in order to meet domestic demand on biofuels^60^.

## Conclusions

In this study, we assessed the potential impacts of climate change on rainfall erosivity over Brazil using bias-corrected baseline (1961–2005) and projected (2007–2099) precipitation data. The latter were taken from downscaled data of two GCMs, HadGEM2-ES and MIROC5, forced by two greenhouses emissions scenarios, RCP4.5 and RCP8.5. The average values of rainfall erosivity of each Brazilian region were calculated and compared with scenario projections.

The average rainfall erosivities during the baseline period were 10,089 ± 3,489 MJ mm ha^−1^ h^−1^ year^−1^ and 10,585 ± 3,420 MJ mm ha^−1^ h^−1^ year^−1^ using estimates from HadGEM2-ES and MIROC5, respectively, compared to 10,437 ± 3,409 MJ mm ha^−1^ h^−1^ year^−1^ form the observed rainfall data. The Northeast, Southeast and South regions are projected to be the most greatly affected regions in terms of rainfall erosivity. The HadGEM2-ES scenarios project a decrease in mean rainfall erosivity in Central-West (−7% to −18%), North (−7% to −20%), Northeast (−1% to −25%), Southeast (−20% to −41%) regions, and both increases and decreases in the South region (−6% to 11%). The MIROC5 scenarios project decreases in mean rainfall erosivity in the Central-West (−5% to −9%), North (−6% to −10%), Northeast (zero to −11%) and Southeast (−5% to −11%) regions, and increases in the South (9% to 33%). It is important to note that the mean values of change in erosivity presented in this paper represent the mean changes in large areas (Brazilian regions), while inside these areas we found both increases and decreases, highlighting the occurrence of spatial variability of rainfall erosivity over Brazil.

Brazil plays an important role in global agricultural production, which will likely be affected by climate change. For example, the possible increase in rainfall erosivity in the South region may affect a significant portion of Brazil’s agricultural production due the increase in soil loss rates and decrease in soil fertility and water availability. On the other hand, the suggested decrease in rainfall erosivity over North and Northeast regions may reinforce the trend of agribusiness development in these areas. To enhance the resilience to climate changes, effective public policies focusing on sustainable agriculture and conservation practices must be implemented, ensuring water and food security. In this study, we present expected or potential trends. We do not intend to predict exactly what will happen quantitatively in the future for any specific piece of agricultural land. More important is that the country, comprising government, industry, farmers, smallholders and society, is prepared for changes in erosivity regardless of the scenarios that may occur.

## Material and Methods

### Study area

Brazil is a continental country (~8,500,000 km^2^) with heterogeneous geographic, climatic and socioeconomic characteristics. Elevation ranges from sea level to up to 2900 m, annual rainfall range from ~400 mm to ~4,000 mm and there are six main biomes (Supplementary Figs S2–S4). In this study, we evaluated the variation of rainfall erosivity over the five geopolitical regions: Central-West (CW), North (N), Northeast (NE), South (S) and Southeast (SE).

### Observed rainfall data

To evaluate and correct the precipitation bias of climate model output, we used a gridded rainfall dataset with 0.25° × 0.25° spatial resolution and 1-month temporal resolution developed by Xavier *et al*.^61^. They used data of ~4,000 rain gauges provided by the Brazilian Water Agency (ANA), the National Institute of Meteorology (INMET), and the Water and Electric Energy Department of São Paulo state (DAEE/SP) from 1980 to 2013. These data are available at: http://careyking.com/data-downloads/.

### Climate change scenarios

**Figure 4:**
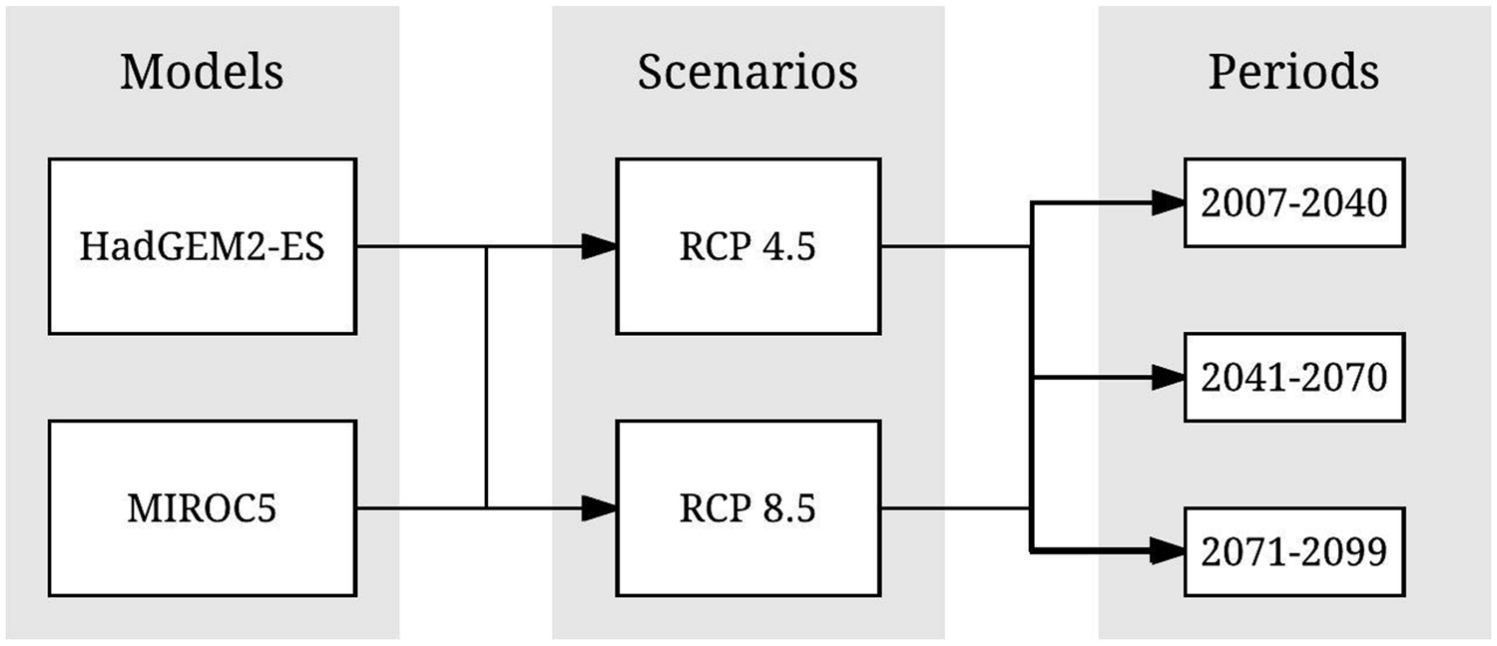
Climate change scenarios used in the present study.

We used baseline (1961–2005) and projected rainfall data that are available from the National Institute of Space Research (INPE) from 2007 to 2099. These data were generated for South-America by the Eta RCM at 0.20° × 0.20° spatial resolution that has been used to downscale output from the two GCMs HadGEM2-ES and MIROC5 forced by two different RCPs (RCP4.5 and RCP8.5)^25, 26^. Data are available at 3-hour, daily and monthly precipitation in future periods: 2007–2040, 2041–2070, and 2071–2099 (Fig. 4).

The RCP scenarios were developed by the research community considering emissions, concentrations and land-use trajectories and are labeled by the expected values of global radiative forcing in 2100^62, 63^. RCP4.5 is considered an intermediate scenario that assumes emissions reduction during the century through the employment of clean technologies and stringent climate policies. This scenario predicts a global forcing radiation of ~4.5 W m^−2^ and concentration of ~650 p.p.m. CO_2_-eq at stabilization after 2100. On the other hand, RCP8.5 is a kind of business-as-usual scenario characterized by no implementation of climate policies, lower rate of technology development, high energy intensity and resilience on fossil fuels that will lead to higher emissions than RCP4.5 over time. Global forcing radiation and atmospheric concentration of CO_2_-eq are projected to reach >8.5 W m^−2^ and >1,370 p.p.m., respectively, in 2100.

### Bias correction of precipitation

To bias correct the projected rainfall data, we used a multiplicative correction (equation 1) that correlated the monthly mean precipitation of the observed and model data in an overlap period (1980–2005), and generated a multiplicative factor (equation 2) for each month at a 0.20° × 0.20° spatial resolution pixel scale. Then, the model data was multiplied by this factor in all periods (baseline and projected) for each time step as shown in Berg *et al*.^64^.

1 $${p}_{iBC}({\rm{t}})={p}_{imodel}(t)\ast f(i)$$

2 $$f(i)=\frac{\overline{{p}_{iobserved}}}{\overline{{p}_{imodel}}}$$

where ${p}_{iBC}$ is the bias corrected monthly precipitation, in mm; ${p}_{imodel}$ is the original model precipitation projection, in mm; $\overline{{p}_{iobserved}}$ is the long-term average of observed precipitation, in mm; $\overline{{p}_{imodel}}$ is the long-term average of model projections of precipitation, in mm; *t* is the time-step (in this case, annual steps); *i* is an index of the month; *f* is the multiplicative factor for each month *i*.

We obtained 12 multiplicative factors, one for each month, using the overlap period (1980–2005) and corrected model precipitation for baseline (1961–2005) and future periods (2007–2040, 2041–2070 and 2071–2099). Results of the bias correction for the overlap period are shown in Supplementary Figs S5 and S6.

### Calculation of the rainfall erosivity index (EI_30_)

The rainfall erosivity index (EI_30_) is determined for individual rainfall events that are classified as erosive (greater than 12.5 mm rainfall depth, or 6.25 mm falling in 15 minutes). Erosivity is analyzed in rain segments of similar intensities by calculating the kinetic energy, as proposed by Wischmeier and Smith^65^ in equation (3):

3 $$e=0.119+0.0873\,lo{g}_{10}\,i$$

where *e* is the kinetic energy (MJ ha^−1^ mm^−1^) and *i* is the average intensity during the time segment (mm h^−1^).

The energy value obtained, *e*, is then multiplied by the amount of rain that fell in the time segment to give the estimate of kinetic energy of the segment. The total kinetic energy of rain (Ect) is obtained by summing the kinetic energies of all the segments. The erosivity index for a storm, EI_30_, is the product of the maximum rain intensity in a continuous 30-minute period of the storm (I_30_) and the computed kinetic energy:

4 $$E{I}_{30}=Ect\,{I}_{30}$$

where *EL*_30_ is the rainfall erosivity index for the individual storm (MJ mm ha^−1^ h^−1^), *Ect* is the total kinetic energy of the rain (MJ ha^−1^) and *I*_30_ is the maximum rain intensity in the 30-minute period (mm h^−1^).

The average annual R-factor, representing the average rainfall erosivity for a year, is obtained from averaging the annual sums of individual EI_30_ erosivity indices (equation 5):

5 $$R=\frac{1}{n}{\sum }_{j=1}^{n}{\sum }_{k=1}^{{m}_{j}}{(E{I}_{30})}_{k}$$

where R is the mean annual rainfall erosivity (MJ mm ha^−1^ h^−1^ year^−1^), *n* is the number of years of data, *m*_*j*_ is the number of erosive events in the *j* year and ${(E{I}_{30})}_{k}$ is the rainfall erosivity index of the *k* ^*th*^ event during the year.

Simplified models that relate the erosivity index to aggregated pluviometric data (e.g., monthly precipitation, annual total precipitation and modified Fournier index) have been proposed to predict rainfall erosivity in regions where sub-daily pluviographic data are not available^28, 66, 67^. Aggregated pluviometric data are generally available in most parts of the world with good spatial and temporal coverage, which is not true for high temporal resolution data with more than 20 years of record needed for the direct calculation of the erosivity index^68–70^. The simplified models are usually generated by regression analysis correlating the EI30 and two independent variables: Modified Fournier Index (MFI) (equation 6) or mean annual precipitation (P)^28^.

6 $$MFI=\frac{{\sum }_{i=1}^{12}\,{{p}_{i}}^{2}}{P}$$

where MFI is the Modified Fournier Index, *p*_*i*_ is the mean monthly precipitation for month *i* (mm) and *P* is the mean annual precipitation (mm).

**Figure 5:**
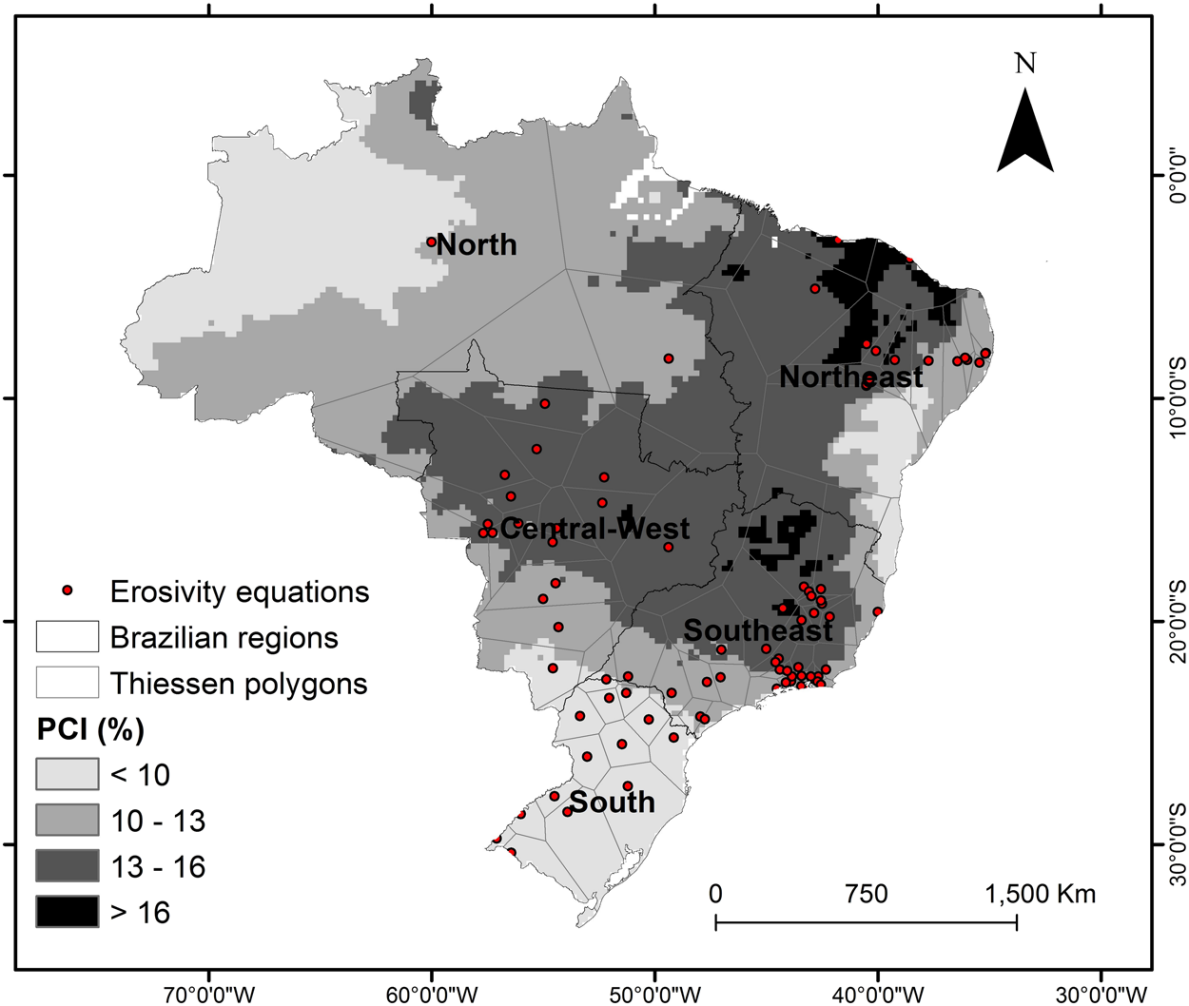
Methodology used to apply the rainfall data to the erosivity index equation. Shades of gray represents the classes of the Precipitation Concentration Index. Light gray lines are the area of influence of each equation. Red points represent the location of development of the erosivity index equations developed in Brazil. Map created with ESRI ArcGIS 10.1 (www.esri.com).

We calculated the R-factor for the past and projected rainfall data using 84 regression equations (See Supplementary Table S1) developed for different regions of Brazil (see Fig. 5). These regression equations come from previous studies that compute EI_30_ using high resolution rainfall data and the equations (3–6). More details about the USLE-R factor regression equations used in this study are discussed in Oliveira *et al*.^28^.

For each equation, we determined an influence area using Thiessen polygons and a Precipitation Concentration Index^71^ that reflects the concentration of the precipitation over the year (equation 7).

7 $$PCI=\frac{{\sum }_{i=1}^{12}\,{p}_{{i}^{2}}}{{({\sum }_{i=1}^{12}P)}^{2}}\times 100$$

where PCI is the Precipitation Concentration Index (%), *p* is the mean precipitation at *i* month (mm) and *P* is the mean annual precipitation (mm).

We applied the observed and projected rainfall data in each equation defined by the influence area and calculated the R-factor pixel by pixel. Then, to generate the erosivity map for each scenario, we converted data in a regular grid with the same spatial resolution of the rainfall data projected by downscaled climate models (0.20° × 0.20°).

### Performance evaluation of estimated rainfall erosivity

To assess the quality of our rainfall erosivity estimations of observed (1980–2013) and baseline (1961–2005) data, we used the coefficient of determination (R^2^), root mean squared error (RMSE) and Nash-Sutcliff efficiency (NSE) (equations 8–10, respectively). To do that, we used rainfall erosivity computed from previous studies using high temporal resolution. These data were provided in Oliveira *et al*.^28^ and updated by Panagos *et al*.^14^.

8 $${R}^{2}=1-[\frac{{\sum }_{i=1}^{n}{({X}_{imod}-{X}_{iobs})}^{2}}{{\sum }_{i=1}^{n}{({X}_{imod}-{X}_{iobs})}^{2}+{\sum }_{i=1}^{n}{({X}_{imod}-{X}_{mean})}^{2}}]$$

9 $$RMSE=\sqrt{\frac{{\sum }_{i=1}^{n}{({X}_{iobs}-{X}_{imod})}^{2}}{n}}$$

10 $$NSE=1-[\frac{{\sum }_{i=1}^{n}{({X}_{iobs}-{X}_{imod})}^{2}}{{\sum }_{i=1}^{n}{({X}_{iobs}-{X}_{mean})}^{2}}]$$

where R^2^ is the coefficient of determination, RMSE is the root mean squared error, NSE is the Nash-Sutcliff efficiency, ${X}_{imod}$ is the estimated rainfall erosivity, ${X}_{iobs}$ is the high-resolution long-term rainfall erosivity and ${X}_{imean}$ is the mean of the high-resolution long-term rainfall erosivity.

### Multitemporal and multiscenario comparison for rainfall erosivity

We used the Student’s t-test with a 95% confidence level to assess if there were significant differences between the bias-corrected mean baseline and projected rainfall erosivities for the HadGEM2-ES and MIROC5 scenarios in the Brazilian regions.

### Data availability

The datasets generated during the present study are available at https://sites.google.com/site/oliveirapts/home.

## Electronic supplementary material

Supplementary Material
