# Supplementary material for: Projected climate change impacts in rainfall erosivity over Brazil
Source: Sci Rep. 2017 Aug 15;7:8130. doi: 10.1038/s41598-017-08298-y (PMC5557879; doi:10.1038/s41598-017-08298-y)
Supplement: Supplementary file 1 — Supplementary Material [file 41598_2017_8298_MOESM1_ESM.pdf]

Projected climate change impacts in rainfall erosivity over Brazil

André Almagro*, Paulo Tarso S. Oliveira, Mark A. Nearing, Stefan Hagemann

*Corresponding author: andre.almagro@gmail.com (+5567981696219)

This file contains:

Supplementary Figure S1

Supplementary Figure S2

Supplementary Figure S3

Supplementary Figure S4

Supplementary Figure S5

Supplementary Figure S6

Supplementary Table S1

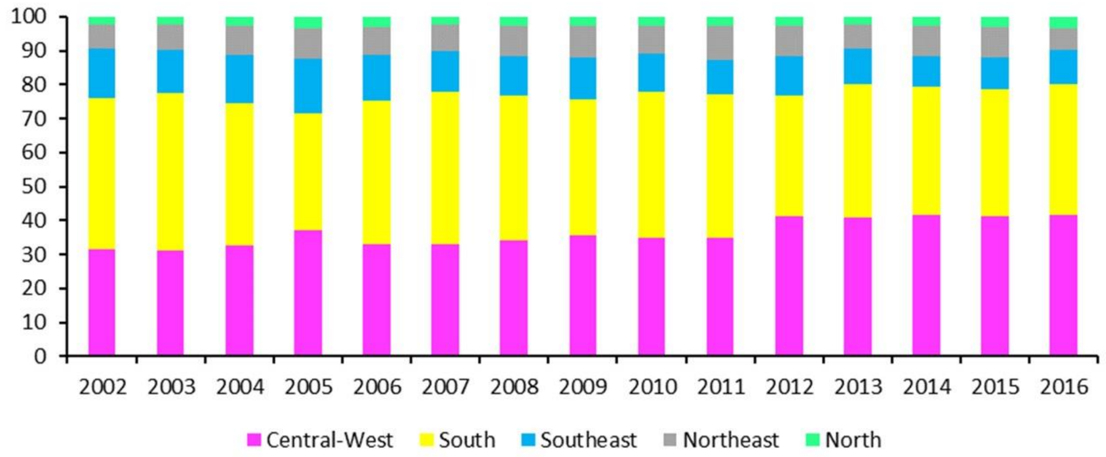

Supplementary Figure S1. Percentage (%) of mass of agricultural production for the Brazilian regions between 2002 and 2016 [1].

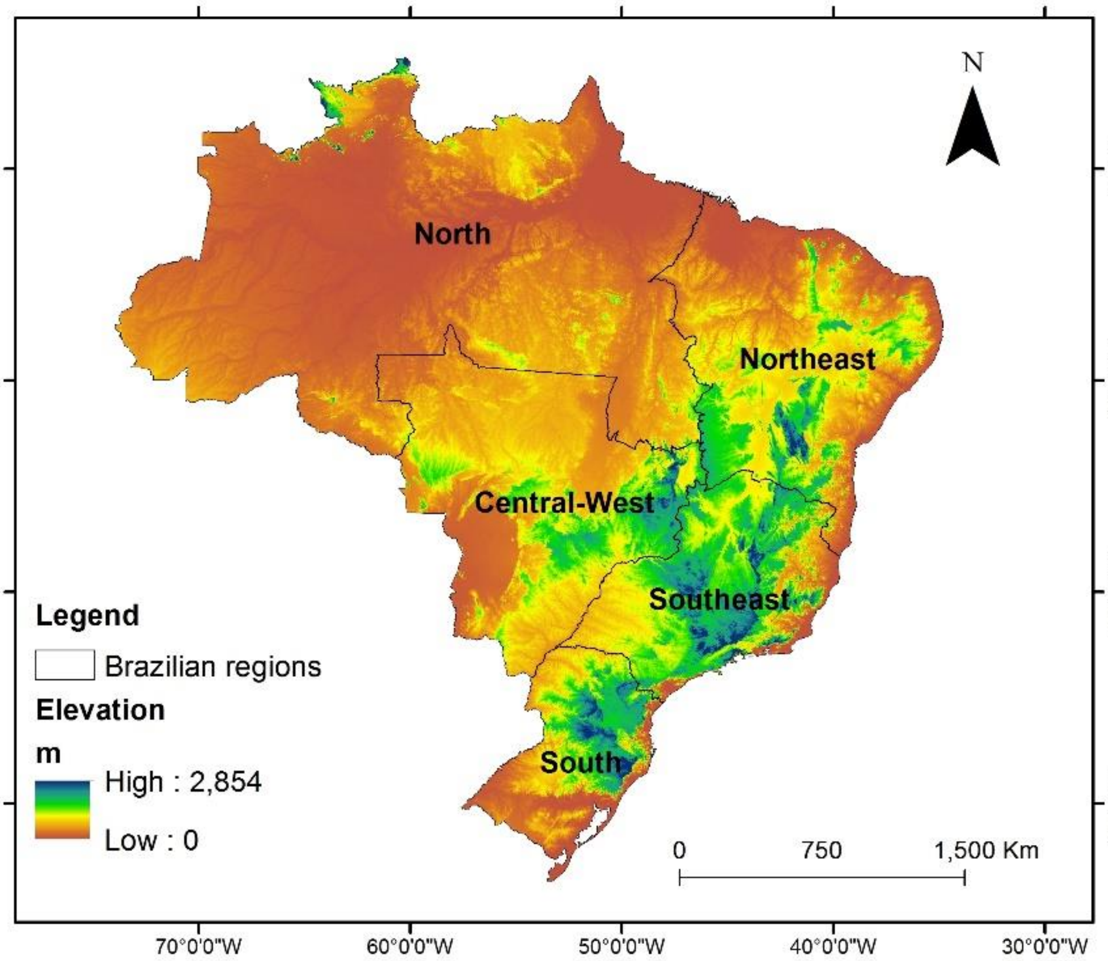

Supplementary Figure S2. Elevation map for Brazil. Map created with ESRI ArcGIS 10.1 (www.esri.com).

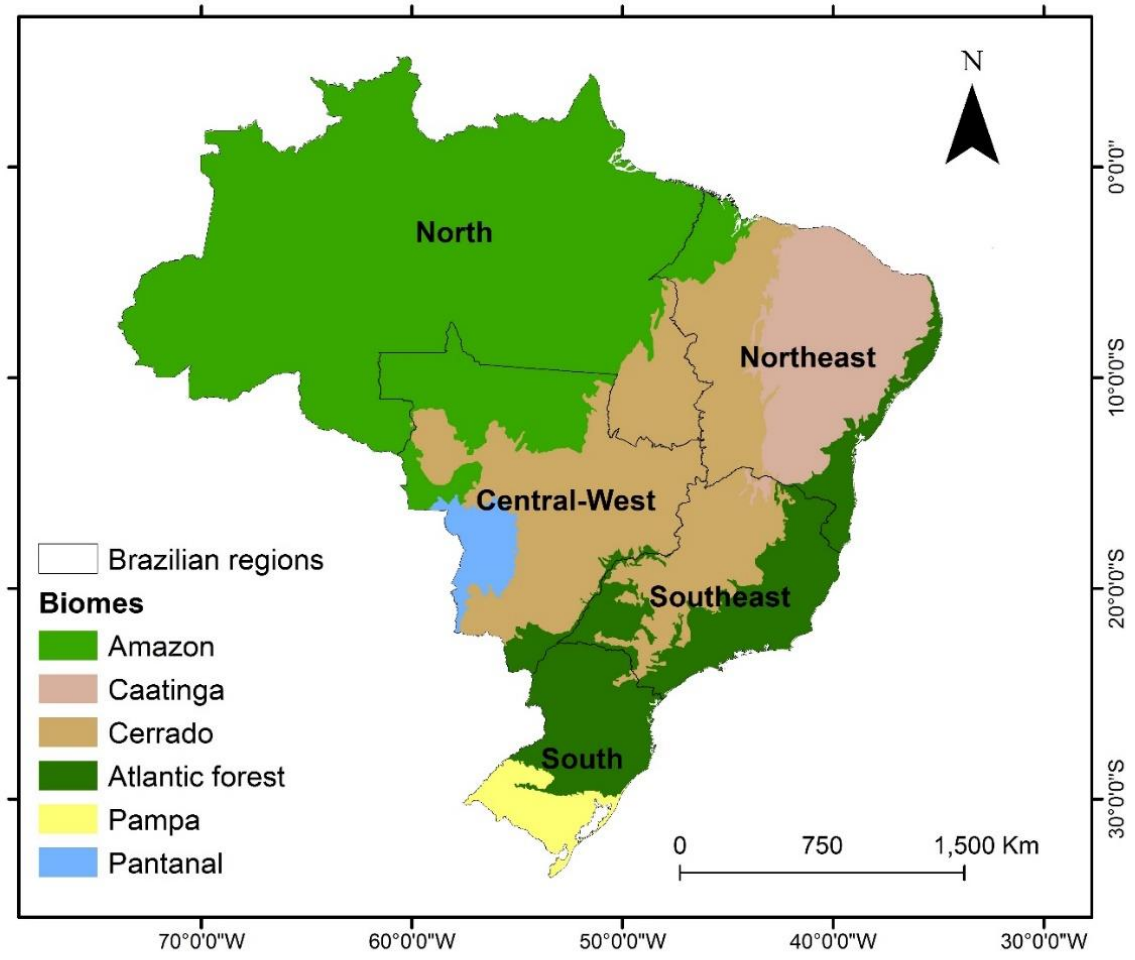

Supplementary Figure S3. Biome map for Brazil. Map created with ESRI ArcGIS 10.1 (www.esri.com).

Brazilian regions

The five geopolitical regions of Brazil: North (N) has a tropical and wet climate, with the highest amount of annual precipitation of all regions (mean ~2,200 mm year^{-1}) (Fig. S4a and S4d); Northeast (NE) has the lowest values of annual precipitation (mean of ~900 mm year^{-1}) and a semi-arid climate (Fig. S4a and S4e); Central-West (CW) is a subtropical climate region with the major part of the annual rainfall concentrated in spring and summer (Fig. S4a and S4c); Southeast (SE) has a subtropical climate with an annual rainfall distribution similar to the Central-West (Fig. S4g); South (S) exhibits the most homogeneous rainfall distribution over the year, and has the lowest temperatures of all regions (Fig. S4f).

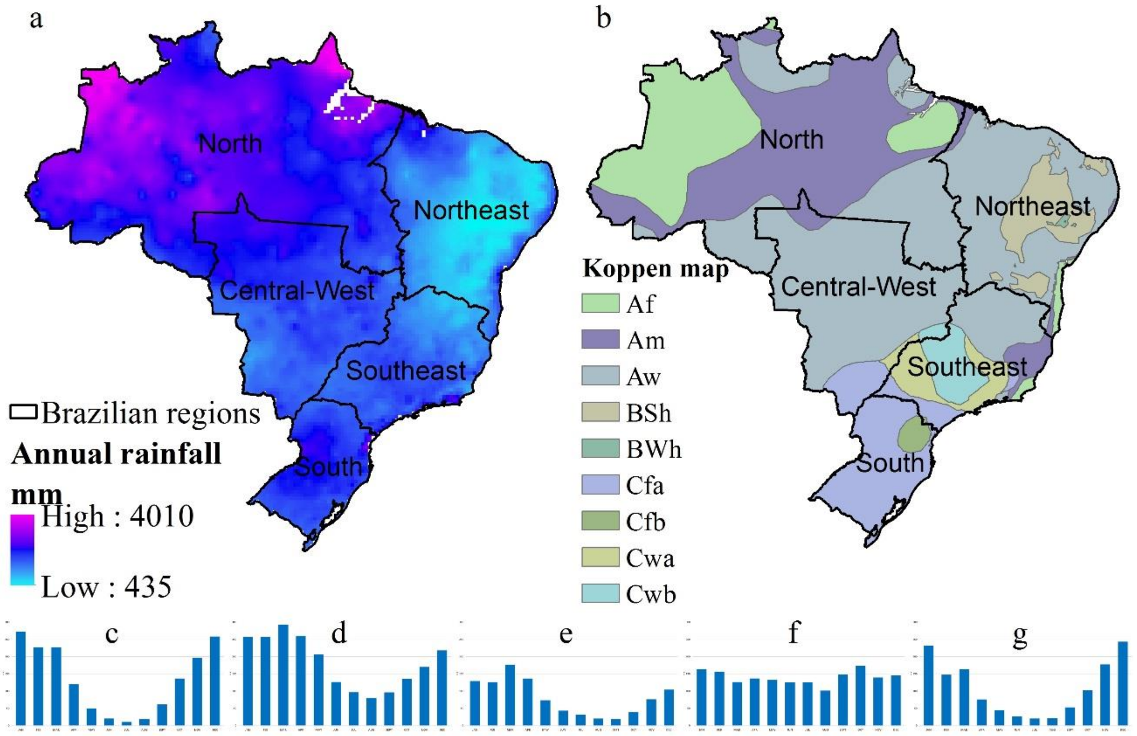

Supplementary Figure S4. Study area characterization. a) Mean annual rainfall of Brazil; b) Köppen climate map of Brazil; c) monthly rainfall distribution in Central-West region; d) monthly rainfall distribution in North region; e) monthly rainfall distribution in Northeast region; f) monthly rainfall distribution in South region; g) monthly rainfall distribution in Southeast region. Maps created with ESRI ArcGIS 10.1 (www.esri.com).

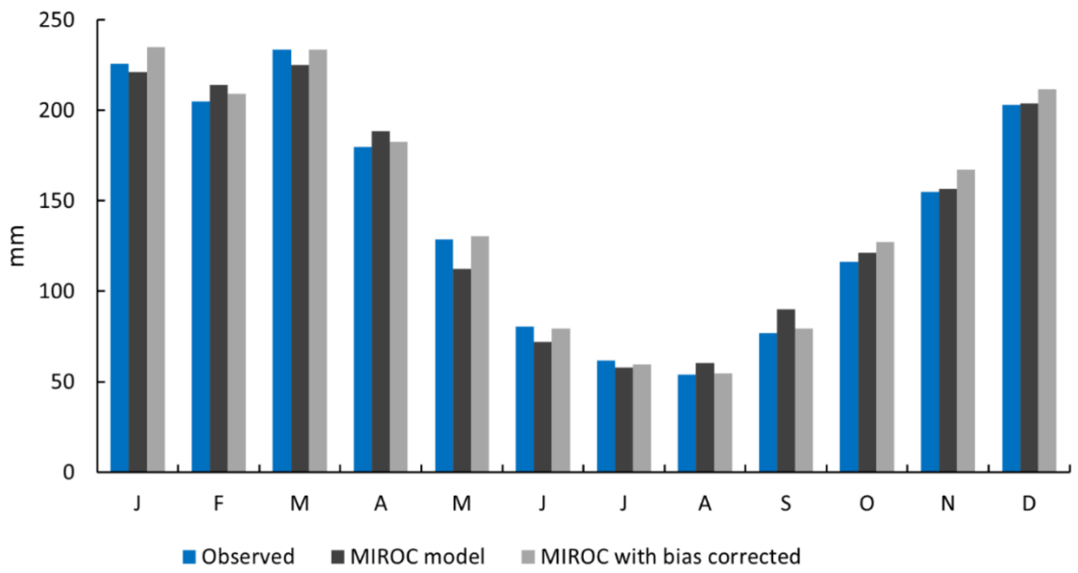

Supplementary Figure S5. Monthly rainfall distribution of the overlap period (1980-2005) for observed data (blue), MIROC original data (black), and bias corrected MIROC data (gray) for Brazil.

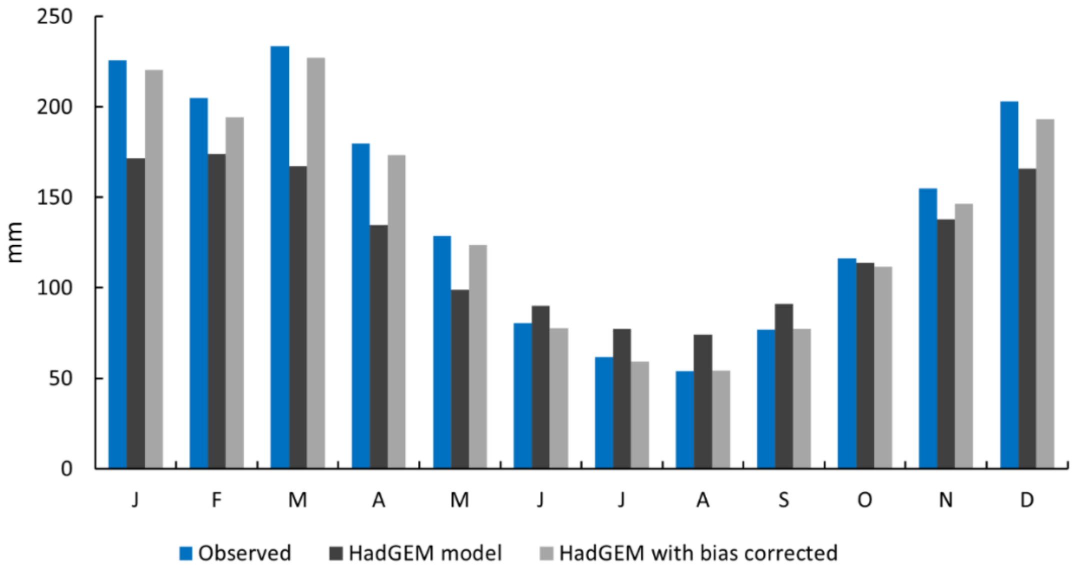

Supplementary Figure S6. Monthly rainfall distribution of the overlap period (1980-2005) for observed data (blue), HadGEM original data (black), and bias corrected HadGEM data (gray) for Brazil.

Supplementary Table S1. Erosivity estimation equations for Brazil.

Longitude	Latitude	City-State	Region	Equation	Author
-60.00	-3.00	Manaus-AM	N	427.7+3.76(MFI)	[2]
-40.51	-9.44	Juazeiro-BA	NE	69.763+42.307(MFI)	[3]
-38.55	-3.73	Fortaleza-CE	NE	$73.989(MFI)^{0.7387}$	[4]
-40.00	-19.58	Aracruz-ES	SE	40.578+7.9075(P)	[5]
-49.38	-16.68	Goiânia-GO	CW	215.33+30.23(MFI)	[6]
-45.00	-21.25	Lavras-MG	SE	$85.672(MFI)^{0.6557}$	[7]
-44.44	-21.68	São Vic. de Minas-MG	SE	$72.8(MFI)^{0.7783}$	[8]
-42.15	-19.80	Caratinga-MG	SE	$321.63(MFI)^{0.48}$	[9]
-44.25	-19.42	Sete Lagoas-MG	SE	$25.3+43.35(MFI)-0.232(MFI)^{2}$	[10]
-42.49	-19.22	Belo Oriente-MG	SE	$215.4(MFI)^{0.65}$	[9]
-43.30	-18.46	Sto. Ant. Itambé-MG	SE	$179.33(MFI)^{0.77}$	[9]
-43.42	-19.96	Santa Bárbara-MG	SE	$170.59(MFI)^{0.64}$	[9]
-42.55	-19.07	Açucena-MG	SE	$158.35(MFI)^{0.85}$	[9]
-42.54	-18.56	Peçanha-MG	SE	-141.07+9.63(P)	[9]
-43.08	-18.67	Sabinópolis-MG	SE	$123.33(MFI)^{0.74}$	[9]
-42.85	-19.64	Antonio Dias-MG	SE	-119.27+7.84(P)	[9]
-42.97	-18.87	Sto. D. do Prata-MG	SE	$114.42(MFI)^{0.81}$	[9]
-44.39	-22.17	Bocaina de Minas-MG	SE	$102.53(MFI)^{0.7586}$	[8]
-54.56	-22.12	Dourados-MS	CW	$80.305(MFI)^{0.8966}$	[11]
-54.32	-20.27	Campo Grande-MS	CW	$139.44(MFI)^{0.6784}$	[11]
-54.45	-18.30	Coxim-MS	CW	$138.33(MFI)^{0.7431}$	[11]
-55.00	-19.00	Coxim-MS	CW	$231(MFI)^{0.570}$	[12]
-57.48	-15.65	Cáceres-MT	CW	$56.115(MFI)^{0.9504}$	[13]
-56.45	-14.41	Diamantino-MT	CW	$51.46(MFI)^{0.883}$	[14]
-55.29	-12.29	Vera-MT	CW	$399.538719(MFI)^{0.458718}$	[15]

Longitude	Latitude	City-State	Region	Equation	Author
-57.27	-16.03	Cáceres-MT	CW	$36.849(MFI)^{1.0852}$	[13]
-52.26	-13.55	Canarana-MT	CW	$317.397829(MFI)^{0.484654}$	[15]
-54.39	-15.84	Poxoréo-MT	CW	$272.865645(MFI)^{0.419164}$	[15]
-57.68	-16.05	Cáceres-MT	CW	$172.6326451(MFI)^{0.5245258}$	[16]
-56.71	-13.44	São J. Rio Claro-MT	CW	$147.262400(MFI)^{0.533025}$	[15]
-54.57	-16.45	Rondonópolis-MT	CW	$133.2004291(MFI)^{0.5372499}$	[17]
-54.92	-10.25	Guarantã do Norte-MT	CW	$115.72(MFI)^{0.746}$	[14]
-56.11	-15.62	Cuiabá-MT	CW	$109.412(MFI)^{0.744}$	[16]
-49.37	-8.23	Conc. de Araguaia-PA	N	321.5+36.2(MFI)	[18]
-40.50	-7.57	Araripina-PE	NE	73.34+23.18(MFI)	[19]
-39.24	-8.28	Cabrobó-PE	NE	73.34+23.18(MFI)	[19]
-40.08	-7.88	Ouricuri-PE	NE	73.34+23.18(MFI)	[19]
-40.50	-9.39	Petrolina-PE	NE	73.34+23.18(MFI)	[19]
-37.72	-8.32	Poço da Cruz-PE	NE	73.34+23.18(MFI)	[19]
-35.98	-8.29	Caruaru-PE	NE	$61.81(MFI)^{0.58}$	[19]
-36.08	-8.19	São Caetano-PE	NE	$61.81(MFI)^{0.58}$	[19]
-36.42	-8.34	Belo Jardim-PE	NE	$61.81(MFI)^{0.58}$	[19]
-35.43	-8.40	Catende-PE	NE	$57.32(MFI)^{0.618}$	[19]
-35.15	-7.98	Olinda-PE	NE	57.25+30.8(MFI)	[19]
-35.18	-8.00	Glória do Goitá-PE	NE	$50.75(MFI)^{0.724}$	[19]
-40.37	-9.15	Bebedouro-PE	NE	$107.96(MFI)^{0.7166}$	[20]
-42.80	-5.09	Teresina-PI	NE	28.133(MFI)+191.58	[21]
-41.78	-2.90	Parnaíba-PI	NE	27.897(MFI)+316.73	[21]
-50.26	-24.41	Centro-Leste Paraná-PR	S	93.29+41.20(MFI)	[22]
-49.14	-25.23	Leste Paraná-PR	S	33.26+40.71(MFI)	[22]
-51.19	-22.48	Norte Paraná-PR	S	216.31+41.30(MFI)	[22]
-52.03	-23.45	Centro Paraná-PR	S	191.79+48.40(MFI)	[22]
-53.34	-24.26	Oeste Paraná-PR	S	182.86+56.21(MFI)	[22]
-51.27	-23.22	Noroeste Paraná-PR	S	164.12+39.44(MFI)	[22]
-53.03	-26.07	Sudoeste Paraná-PR	S	144.86+55.20(MFI)	[22]
-51.46	-25.52	Cento-Sul Paraná-PR	S	107.52+46.89(MFI)	[22]
-43.88	-22.70	Piraí-RJ	SE	95.40+22.69(MFI)	[23]
-43.28	-22.96	Rio de Janeiro-RJ	SE	-76.27+53.31(MFI)	[23]
-44.52	-23.03	Angra dos Reis-RJ	SE	73.21+44.61(MFI)	[23]
-42.32	-22.17	Nova Friburgo-RJ	SE	67.991+33.856(MFI)	[24]
-43.41	-22.46	Seropédica-RJ	SE	64.866+38.138(MFI)	[24]
-42.94	-22.58	Magé-RJ	SE	64.59+47.68(MFI)	[23]
-43.84	-22.48	Barra do Piraí-RJ	SE	50.36+24.53(MFI)	[23]
-42.66	-22.48	Conc. de Macabu-RJ	SE	39.86+37.90(MFI)	[23]
-42.70	-22.71	Rio Bonito-RJ	SE	38.48+35.13(MFI)	[23]
-43.42	-22.92	Rio de Janeiro-RJ	SE	3.89+37.76(MFI)	[23]
-44.58	-21.84	Carmo-RJ	SE	223.87+21.00(MFI)	[23]
-44.06	-22.23	Valença-RJ	SE	194.08+27.74(MFI)	[23]
-43.00	-22.48	Magé-RJ	SE	146.28+46.37(MFI)	[23]
-42.55	-22.85	Saquarema-RJ	SE	-13.36+50.02(MFI)	[23]

Longitude	Latitude	City-State	Region	Equation	Author
-44.12	-22.75	Rio Claro-RJ	SE	118.71+38.48(MFI)	23
-43.56	-22.07	Rio das Flores-RJ	SE	112.54+20.70(MFI)	23
-56.00	-28.65	São Borja-RS	S	$55.564(MFI)^{1.1054}$	25
-56.43	-30.38	Quaraí-RS	S	-47.35+82.72(MFI)	26
-57.08	-29.75	Uruguaiana-RS	S	$-252.559+117.069(MFI) -1.657(MFI)^2$	27
-54.48	-27.85	Santa Rosa-RS	S	$118.52(MFI)^{0.8034}$	28
-53.90	-28.55	Ijuí-RS	S	$109.65(MFI)^{0.76}$	29
-51.20	-27.40	Campos Novos-SC	S	$59.265(MFI)^{1.087}$	30
-49.23	-23.22	Piraju-SP	SE	$72.5488(MFI)^{0.8488}$	31
-47.04	-22.52	Campinas-SP	SE	$68.730(MFI)^{0.841}$	32
-47.95	-24.28	Sete Barras-SP	SE	316.20+55.40(MFI)	33
-47.75	-24.40	Juquiá-SP	SE	207.21+40.65(MFI)	33
-47.01	-21.28	Mococa-SP	SE	$111.173(MFI)^{0.691}$	34
-52.17	-22.62	Teodoro Sampaio-SP	SE	106.8183+46.9562(MFI)	35

REFERENCES

1. IBGE. Levantamento Sistematico Da Produção Agrícola. *Novembro 2016* **29,** 1–85 (2016).

2. Oliveira Jr., R. C. & Medina, B. F. A erosividade das chuvas em Manaus (AM). *Rev. Bras. Ciência do Solo* **14,** 235–239 (1990).

3. Silva, A. M. Rainfall erosivity map for Brazil. *Catena* **57,** 251–259 (2004).

4. Dias, A. S. & Silva, J. R. C. A erosividade das chuvas em Fortaleza (CE). I - Distribuição, probabilidade de ocorrência e período de retorno - 1ª Aproximação. *Rev. Bras. Ciencias do Solo* **27,** 335–345 (2003).

5. Martins, S. G., Avanzi, J. C. & Naves, M. L. Rainfall erosivity and rainfall return period in the experimental watershed of Aracruz, in the coastal plain of Espírito Santo, Brazil. *Rev. Bras. Ciência do Solo* **34,** 999–1004 (2010).

6. Silva, M. L. N., De Freitas, P. L., Blancaneaux, P. & Curi, N. Índices de erosividade das chuvas da região de Goiânia, GO. *Pesqui. Agropecu. Bras.* **32,** 977–985 (1997).

7. Aquino, R. F. *et al.* Erosividade das chuvas e tempo de recorrência para lavras, Minas Gerais. *Rev. Ceres* **61,** 9–16 (2014).

8. Mello, C. R., Norton, L. D., Curi, N. & Yanagi, S. N. M. Sea Surface Temperature (SST) and rainfall erosivity in the Upper Grand River Basin, Southeast Brazil. *Cienc. e Agrotecnologia* **36,** 53–59 (2012).

9. Silva, R. B., Iori, P., Armesto, C. & Bendini, H. N. Assessing Rainfall Erosivity with Artificial Neural Networks for the Ribeira Valley, Brazil. *Int. J. Agron.* **2010,** 1–7 (2010).

10. Marques, J. J. G. S. M., Alvarenga, R. C., Curi, N. & Santana, D. P. Índices de erosividade da chuva, perdas de solo e fator erodibilidade para dois solos da região dos Cerrados - primeira aproximação. *Rev. Bras. Ciência do Solo* **21,** 427–434 (1997).

11. Oliveira, P. T. S. de, Rodrigues, D. B. B., Alves Sobrinho, T., de Carvalho, D. F. & Panachuki, E. Spatial variability of the rainfall erosive potential in the state of Mato Grosso do Sul, Brazil. *Eng. Agríc. Jaboticabal* **32,** 69–79 (2012).

12. Machado, D. O., Alves Sobrinho, T., Ribeiro, A. D. S., Ide, C. N. & Oliveira, P. T. S. Rainfall erosivity for Pantanal biome. *Eng. Sanit. e Ambient.* **19,** 195–201 (2014).

13. Morais, L. F. . *et al.* Índice EI30 e sua Relação com o Coeficiente de Chuva do Sudoeste do Mato Grosso. *Rev. Bras. Ciencias do Solo* **15,** 339–344 (1991).

14. Almeida, C. O. S. Erosividade das chuvas no estado de Mato Grosso. *Master's thesis* (2009).

15. Almeida, C. O. S., Amorim, R. S. S., Eltz, F. L. F., Couto, E. G. & Jordani, S. a. Erosividade da chuva em municípios do Mato Grosso: Distribuição sazonal e correlações com dados pluviométricos. *R. Bras. Eng. Agríc. Ambient.* **16,** 142–152 (2012).

16. Almeida, C. O. S., Amorim, R. S. S., Couto, E. G., Eltz, F. L. F. & Borges, L. E. C. Potencial erosivo da chuva de Cuiabá, MT: distribuição e correlação com a precipitação pluviométrica. *Rev. Bras. Eng. Agrícola e Ambient.* **15,** 178–184 (2011).

17. Almeida, C. O. S., Amorim, R. S. S., Eltz, F. L. F., Couto, E. G. & Pelissari, A. L. Correlação do índice de erosividade (EI30) com o coeficiente de chuvas em Cáceres (MT) e Rondonópolis (MT). *Proc. XXXIII Congr. Bras. Ciência do Solo* 1–4 (2011). doi:10.1017/CBO9781107415324.004

18. Oliveira Jr., R. C. Índice de Erosividade das Chuvas na Região de Conceicão do Araguaia, Pará. *EMBRAPA-CPATU* **165,** 20 (1996).

19. Cantalice, J. R. B., Bezerra, S. A., Figueira, S. B., Inácio, E. D. S. B. & Silva, M. D. R. D. O. Linhas Isoerosivas Do Estado De Pernambuco - 1ª Aproximação. *Rev. Caatinga* **22,** 75–80 (2009).

20. Silva, M. T., Silva, V. P. R., Souza, E. P., Araújo, A. L. & Azevedo, J. V. V. in *Recur. Nat. do Semiárido – Estud. Apl.* 325–339 (2016). doi:10.13140/RG.2.1.3516.3766

21. Santos Júnior, A. B. Erosividade e padrão de chuvas em Teresina e Parnaíba no Piauí. (2011).

22. Rufino, R. L., Biscaia, R. C. M. & Merten, G. H. Determinação do potencial erosivo da chuva do Estado do Paraná através da pluviometria: terceira aproximação. *Rev. Bras. Ciência do Solo* **17,** 439–444 (1993).

23. Gonçalves, F. a., Silva, D. D. Da, Pruski, F. F., Carvalho, D. F. De & Cruz, E. S. Da. Índices e espacialização da erosividade das chuvas para o Estado do Rio de Janeiro. *Rev. Bras. Eng. Agrícola e Ambient.* **10,** 269–276 (2006).

24. Carvalho, D. F. De, Montebeller, C. a., Bertol, I., Valcarcel, R. & Franco, E. M. Rainfall patterns and erosion indices at Seropedica and Nova Friburgo, Rio de Janeiro - Brazil. *Rev. Bras. Eng. Agrícola e Ambient.* **9,** 7–14 (2005).

25. Cassol, E. A. *et al.* Erosividade, padrões hidrológicos, período de retorno e probabilidade de ocorrência das chuvas em São Borja, RS. *Rev. Bras. Cienc. do Solo* **32,** 1239–1251 (2008).

26. Bazzano, M. G. P., Eltz, F. L. F. & Cassol, E. A. Erosividade, coeficiente de chuva, padrões e período de retorno das Chuvas de Quaraí, RS. *Rev. Bras. Cienc. do Solo* **31,** 1205–1217 (2007).

27. Hickmann, C., Eltz, F. L. F., Cassol, E. A. & Cogo, C. M. Erosividade das chuvas em Uruguaiana, RS, determinada pelo índice EI30, com base no período de 1963 a 1991. *Rev. Bras. Ciência do Solo* **32,** 825–831 (2008).

28. Mazurana, J., Cassol, E. A., Santos, L. C., Eltz, F. L. F. & Bueno, A. C. Erosividade, padrões hidrológicos e período de retorno das chuvas erosivas de Santa Rosa (RS). *Rev. Bras. Eng. Agrícola e Ambient.* **13,** 975–983 (2009).

29. Cassol, E. A., Martins, D., Luiz, F., Eltz, F. & Lima, V. S. De. Erosivity and hydrological patterns of Ijui (RS, Brazil) rainfalls in the period of 1963 to 1993. *Rev. Bras. Agrometeorol.* **15,** 220–231 (2007).

30. Bertol, I. Evaluation for rain erosivity for Campos Novos (SC) during the 1981-1990 period. *Pesqui. Agropecuária Bras.* **29,** 1453–1458 (1994).

31. Roque, C. G., Carvalho, M. P. & Prado, R. M. Fator erosividade da chuva de Piraju (SP): distribuição, probabilidade de ocorrência, período de retorno e correlação com o coeficiente de chuva. *Rev. Bras. Ciência do Solo* **25,** 147–156 (2001).

32. Lombardi Neto, F. & Moldenhauer, W. C. Erosividade da chuva: sua distribuicao e relacao com as perdas de solo em Campinas (SP). *Bragantia* **51,** 189–196 (1992).

33. Silva, R. B., Iori, P. & Silva, F. A. D. M. Proposition and compare of equations to estimate the rainfall erosivity in two cities of São Paulo state. *Irriga* **14,** 533–547 (2009).

34. Carvalho, M. P. E., Lombardi Neto, F., Vasques Filho, J. & Catâneo, A. Correlação entre o índice de erosividade EI30 médio mensal e o coeficiente de chuva do município de Mococa, SP. *Científica - Rev. Agron.* **19,** 1–7 (1991).

35. Colodro, G., Carvalho, M. P., Roque, C. G. & Prado, R. M. Rainfall erosivity: its distribution and relationship with the nonrecording rain gauge precipitation at Teodoro Sampaio, São Paulo, Brazil. *Rev. Bras. Ciência do Solo* **26,** 809–818 (2002).
